# Limited value of serum neurofilament light chain in diagnosing amyotrophic lateral sclerosis

**DOI:** 10.1093/braincomms/fcad163

**Published:** 2023-05-19

**Authors:** Jennifer C Davies, Thanuja Dharmadasa, Alexander G Thompson, Evan C Edmond, Katie Yoganathan, Jiali Gao, Kevin Talbot, Martin R Turner

**Affiliations:** Nuffield Department of Clinical Neurosciences, University of Oxford, Oxford, OX3 9DU, UK; Nuffield Department of Clinical Neurosciences, University of Oxford, Oxford, OX3 9DU, UK; Nuffield Department of Clinical Neurosciences, University of Oxford, Oxford, OX3 9DU, UK; Nuffield Department of Clinical Neurosciences, University of Oxford, Oxford, OX3 9DU, UK; Nuffield Department of Clinical Neurosciences, University of Oxford, Oxford, OX3 9DU, UK

**Keywords:** neurofilament light chain, biomarker, diagnosis, prognosis

## Abstract

A biomarker specific for the diagnosis of amyotrophic lateral sclerosis must be sensitive across a spectrum of clinical heterogeneity. Neurofilament light chain levels in amyotrophic lateral sclerosis correlate with the rate of disability progression. Previous attempts to establish a diagnostic role for neurofilament light chain have been limited to comparison with healthy individuals or controls with alternative diagnoses unlikely to be confused with amyotrophic lateral sclerosis in real-world clinical practice.

In a tertiary amyotrophic lateral sclerosis referral clinic, at first visit, serum was taken for neurofilament light chain measurement after prospectively recording the clinical diagnosis as ‘amyotrophic lateral sclerosis’, ‘primary lateral sclerosis’, ‘alternative’ or ‘currently uncertain’.

Of 133 referrals, 93 patients were diagnosed with amyotrophic lateral sclerosis (median neurofilament light chain 218.1 pg/ml, interquartile range 130.7–311.9), three primary lateral sclerosis (65.6, 51.5–106.9) and 19 alternative diagnoses (45.2, 13.5–71.9) at first visit. Of 18 initially uncertain diagnoses, eight were subsequently diagnosed with amyotrophic lateral sclerosis (98.5, 45.3–300.1). Neurofilament light chain ≥110.9 pg/ml had a positive predictive value of 0.92 for amyotrophic lateral sclerosis; <110.9 pg/ml had a negative predictive value of 0.48.

In a specialized clinic, neurofilament light chain is largely confirmatory to clinical judgement in diagnosing amyotrophic lateral sclerosis and has limited ability to exclude alternative diagnoses. The current, important, value of neurofilament light chain is its potential to stratify patients with amyotrophic lateral sclerosis by disease activity and as a biomarker in therapeutic trials.

## Teaser

To consider any added value of serum neurofilament light chain to the routine amyotrophic lateral sclerosis diagnostic pathway, Davies *et al.* prospectively analysed tertiary clinic referrals. In this setting, neurofilament light was neither a reliable rule-in nor rule-out test for amyotrophic lateral sclerosis or any alternative diagnosis.

## Graphical Abstract

**Graphical abstract:**
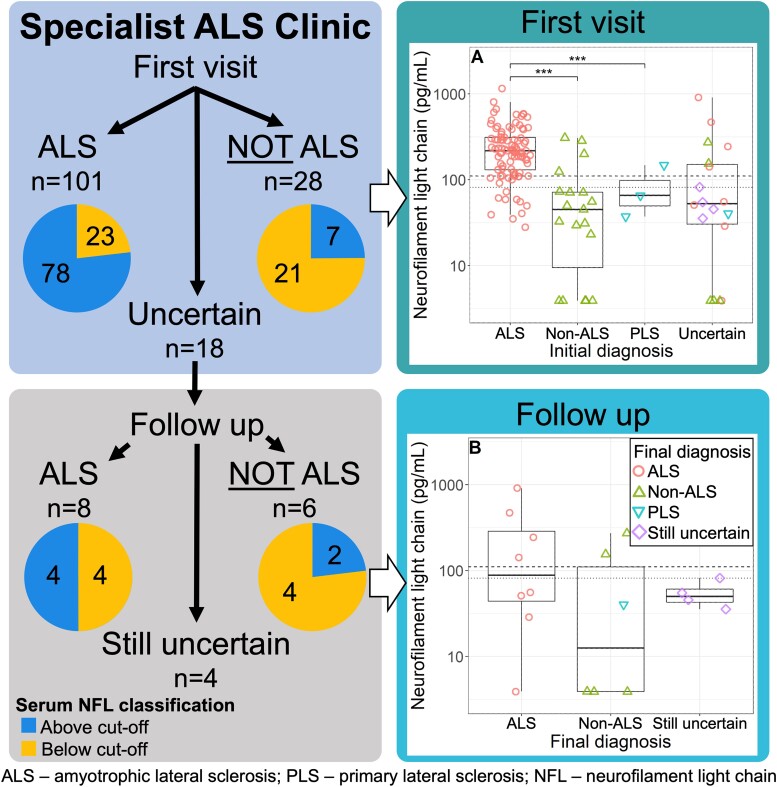

## Introduction

In most cases, the presence of characteristic clinical features of typical amyotrophic lateral sclerosis (ALS)—progressive motor weakness accompanied by clinical or neurophysiological evidence of lower and upper motor neuron dysfunction—makes the diagnosis straightforward, and there are few plausible mimic disorders.^1^ ALS is clinically heterogeneous, however. A significant proportion of cases have atypical features such as markedly slower progression of weakness, often in the context of prolonged sub-regional restriction, or a predominance of lower or upper motor neuron signs. Furthermore, comorbidities, e.g. spinal spondylosis, may interfere with the interpretation of clinical signs.^2–5^ The average time of one year from first symptoms to a diagnosis of ALS is driven by factors beyond the lack of a highly specific diagnostic test.^6,7^ Reasons for diagnostic delay include the insidious onset of weakness in ALS and the delay in primary care to recognize dysfunction as neurological, with the risk of misdirected referral and inappropriate spinal surgery in some cases. A diagnostic biomarker for ALS for use in population screening remains an aspiration. More realistically, using biomarkers to remove diagnostic doubt at the point of clinical suspicion would be a major advance allowing earlier and possibly more inclusive enrollment to therapeutic trials in ALS, as well as optimizing care-planning. Equally valuable for ALS therapeutic development would be a biomarker that reflects individual disease activity to provide objective evidence of positive disease modification.

Neurofilaments are intermediate filament proteins that are a major structural constituent of axons of the central and peripheral nervous system. Since the first observation of elevated levels of neurofilaments in the cerebrospinal fluid (CSF) of ALS patients,^8^ extensive work has demonstrated that neurofilament levels, most consistently neurofilament light chain (NfL), are closely associated with measures of disease aggressiveness such as survival and rate of disability progression. Developments in assay technology have now improved the accuracy of NfL measurement in blood compared with CSF leading to their adoption as a secondary outcome measures in ALS clinical trials.^9–12^

The performance of NfL levels in distinguishing ALS from non-ALS blood and CSF samples has been studied extensively. Despite elevated neurofilament levels being a feature of many neurological diseases,^13^ they have shown encouraging results in rapidly progressive conditions such as ALS where levels are more consistently high. Plasma and serum NfL have been reported to have a diagnostic sensitivity ranging from 76–100% and a specificity of 75–92%.^14–17^ However, studies have typically compared ALS patient samples either with those from predominantly healthy controls or patients retrospectively labelled as having other neurological conditions, many of which would be readily distinguished from ALS on clinical grounds alone.

This study applied a prospective approach to consider the diagnostic utility of serum NfL level taken at the first visit to a tertiary referral clinic in relation to a clinically based diagnosis.

## Materials and methods

### Participants

All symptomatic patients attending the ALS tertiary referral clinic at the John Radcliffe Hospital in Oxford for the first time between August 2020 and September 2021 were invited to give a blood sample ahead of their clinical assessment after providing written consent for ‘A Prospective Study of Neurological Biomarkers’ (Research Ethics Committee approval reference 20/WA/0027). Based on an experienced ALS neurologist’s clinical assessment (M.R.T., K.T. and A.G.T.), participants were assigned one of the following diagnostic categories: (i) ALS (within Gold Coast criteria^18^), (ii) primary lateral sclerosis (PLS) (within Philadelphia criteria^19^), (iii) a specific alternative diagnosis or, (iv) uncertain diagnosis. Patients in the latter group were re-categorized once a diagnosis became established during routine clinic follow-up.

Clinical parameters, including site of symptom onset and revised ALS functional rating scale score, were obtained by retrospective review of the clinical notes. Disease progression rate was calculated using the formula (48-revised ALS functional rating scale score)/(months from onset of first weakness).

### Serum NfL measurements

Serum was obtained from peripheral blood in BD vacutainer SSTII advance tubes and centrifuged at 3500 rpm for 10 minutes. Samples were processed within 2 hours of collection and stored at −80°C until measurement. First freeze-thaw serum NfL was measured in duplicate using the R-plex NfL Meso Scale Discovery assay, according to the manufacturer’s instructions. The limit of detection (LOD) for the assay is 5.5 pg/ml with a working range of 12–50 000 pg/ml. For NfL levels below the linear range of detection, the equation LOD/√2 was applied, and a substitution value of 3.9 pg/ml was applied.^20^ The intra-assay coefficients of variation were <15%, and the inter-assay coefficients of variation were 3.5% and 6.5% for two pooled samples (*n* = 4 for each).

### Statistical analysis

Statistical analysis was performed using *R*. NfL levels did not follow a normal distribution, so log_10_ transformation and non-parametric analysis were applied. For two or more groups, homogeneity of variance across groups was first tested using Levene’s test. Where the assumption of equal variances was violated, Welch’s ANOVA was used. Dunn’s multiple comparison test was then performed, and pairwise comparisons were adjusted by Bonferroni method. Sensitivity, specificity, and positive and negative predictive values were calculated, firstly using a prespecified cut-off corresponding to the 90th percentile of healthy control values, then using a cut-off corresponding to the midpoint of the range of NfL cut-offs with maximal Youden’s index. In calculations of sensitivity, specificity, positive and negative predictive value, healthy participants were excluded, and patients diagnosed with PLS were grouped with patients with non-ALS diagnoses. Exact confidence intervals were calculated using the epiR package.

## Results

### Demographic data

Demographic data and NfL concentrations are given in Table 1. Ninety-three patients were diagnosed with ALS, three with PLS and 19 with an alternative diagnosis at the first visit (Supplementary table 1). The diagnosis in 18 patients was not clear at the first visit, of whom eight were diagnosed with ALS, one with PLS and five with a non-ALS diagnosis at a subsequent visit. In four patients, the clinical picture did not permit distinction between ALS and an alternative diagnosis at the time of analysis (7–14 months from their initial visit).

**Table 1:** Clinical and demographic characteristics

	Diagnosed at first visit			Diagnosis uncertain at first visit		
Diagnosis at first visit	ALS	PLS	Non-ALS	Uncertain →	Uncertain →	Uncertain →
				ALS	non-ALS^a^	uncertain^b^
*n*	93	3	19	8	6	4
Female (%)	35 (37.6)	0 (0.0)	3 (15.8)	1 (12.5)	0 (0.0)	1 (25.0)
Age at first visit (years), median (IQR)	65.0 (55.0–71.0)	57.0 (52.0–59.5)	58.0 (49.0–71.0)	61.5 (58.5–71.2)	57.0 (53.8–58.8)	62.0 (55.5–66.5)
Age at symptom onset (years), median (IQR)	64.2 (54.1–70.5)	51.2 (46.5–55.7)	53.1 (44.9–67.9)	61.2 (57.9–71.5)	54.6 (51.8–57.4)	58.0 (52.4–62.8)
Disease duration (months), median (IQR)	13.1 (8.9–22.4)	64.5 (44.3–67.7)	11.5 (6.6–23.7)	12.6 (5.6–17.4)	27.4 (23.9–35.1)	42.9 (30.7–50.9)
Diagnostic latency (months), median (IQR)	11.5 (7.4–19.6)	64.5 (44.3–67.7)	7.1 (6.0–162.8)	14.2 (8.3–29.5)	44.1 (31.3–46.1)	52.3 (37.1–63.0)
Bulbar onset (%)	27 (29.0)	0 (0.0)	0 (0.0)	3 (37.5)	2 (33.3)	0 (0.0)
ALSFRS-R at first visit (points), median (IQR)	41.0 (37.0–43.0)	–	–	43.5 (42.0–44.2)	–	–
Disease progression rate at first visit (points/month), median (IQR)	0.6 (0.3–1.0)	–	–	0.5 (0.3–0.8)	–	–
Serum NfL (pg/ml), median (IQR)	218.1 (130.7–311.9)	65.6 (51.4–106.8)	45.2 (13.5–71.8)	98.5 (45.3–300.1)	22.1 (3.9–125.8)	50.2 (43.1–61.6)

Data are given as median (IQR).

a Includes one patient initially with an uncertain diagnosis who was diagnosed with PLS during follow up.

b Diagnosis not confirmed after 7–14 months from first visit at the tertiary clinic.

ALSFRS-R = revised ALS functional rating scale score.

### Diagnostic performance of serum neurofilament light chain

**Figure 1:**
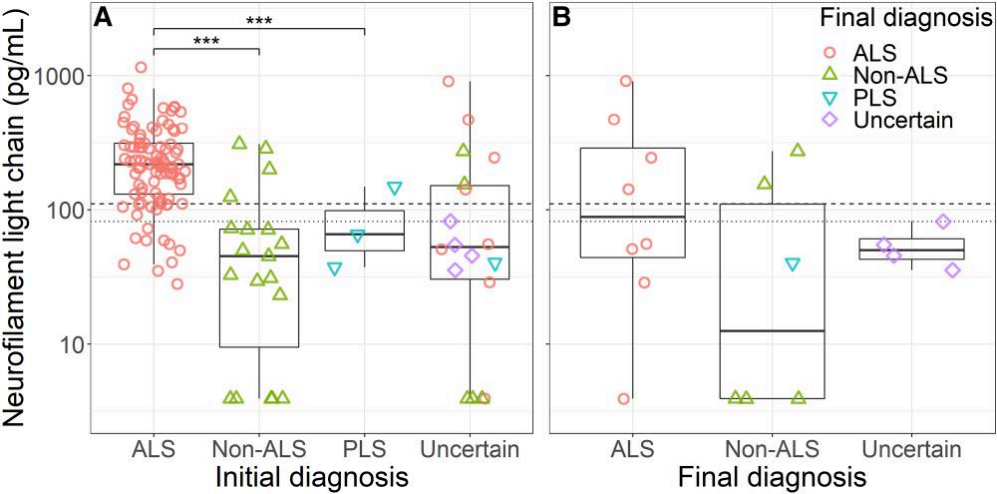
**Serum NfL levels of patients referred to the tertiary ALS clinic.** (**A**) Serum NfL levels and diagnostic categories at the first visit. Levels of serum NfL were significantly elevated in ALS patients compared with those with a non-ALS diagnosis or PLS. (**B**) Serum NfL levels in patients with uncertain diagnosis who were subsequently diagnosed with ALS or received a non-ALS diagnosis during follow up, or in whom it was not possible to distinguish between ALS and a non-ALS diagnosis at the end of follow up. *P*-values are given for Welch’s ANOVA of log-transformed levels, with Dunn’s multiple comparison adjustment. Dashed line indicates 90th centile of healthy control values, and dotted line indicates mid-point of serum NfL range where Youden’s index is maximal. *** *P* < 0.001. ALS amyotrophic lateral sclerosis, PLS primary lateral sclerosis.

Levels of serum NfL were significantly elevated in ALS patients compared with those given an alternative diagnosis or diagnosed with PLS at the first visit (ALS median 218.1 pg/ml, IQR 130.7–311.9; PLS 65.6 pg/ml, 51.5–106.9; non-ALS 45.1 pg/ml, 13.5–71.9 pg/ml; ALS versus PLS *P* < 0.001; ALS versus non-ALS *P* < 0.001; Fig. 1A).

Using a prespecified cut-off of 110.9 pg/ml, corresponding to the 90th percentile of healthy control values, diagnostic accuracy of serum NfL was calculated for all patients referred (excluding participants for whom the diagnosis remained undetermined at the end of follow up). This correctly classified 78/101 ALS patients and 21/28 non-ALS patients giving a sensitivity of 0.77 (95% confidence interval (CI) 0.68–0.85) and a specificity of 0.75 (CI 0.55–0.89), corresponding to a positive predictive value of 0.92 (CI 0.84–0.97) and negative predictive value of 0.48 (CI 0.32–0.63).

In the group with an uncertain diagnosis at first visit, NfL correctly classified 4/8 ALS patients and 4/6 patients with an alternative diagnosis. Four patients remained undiagnosed at the end of follow up, all below the cut-off.

The ALS patients with NfL below the cut-off had less aggressive disease, with lower disease progression rate (median: below cut-off 0.21 points per month, above cut-off 0.29 points per month, *P* = 0.029) and longer latency to diagnosis than those with NfL above the cut-off (median: latency below cut-off 55.0 months, above cut-off 19.0 months, *P* < 0.001). Those above the cut-off were of older age (median: below cut-off 58.0 years, above cut-off 54.5 years, *P* = 0.015). A greater proportion of ALS cases with low NfL were of spinal onset, though this was not statistically significant (below cut-off 23/27 versus above cut-off 48/74; odds ratio (OR) 3.13, *P* = 0.053, CI 0.92–14.29).

To investigate the performance of an alternative optimized cohort-specific cut-off, we analysed performance using a cut-off corresponding to the midpoint of the range of NfL cut-offs with maximal Youden’s index (82.0 pg/ml). This yielded a sensitivity of 0.86 (CI 0.78–0.92) and a specificity of 0.75 (CI 0.55–0.89), with positive predictive value of 0.93 (CI 0.85–0.97) and negative predictive value of 0.60 (CI 0.42–0.76), correctly identifying 87/101 ALS patients and 21/28 patients with non-ALS diagnoses. In the group with an uncertain diagnosis at the first visit, this cut-off correctly classified 4/8 ALS and 4/6 non-ALS patients.

## Discussion

This study used a prospective approach to try to consider the independent value of blood NfL in the diagnosis of ALS versus an alternative diagnosis. The majority of patients referred to our clinic are ultimately diagnosed with ALS, with most having been referred by neurologists with this diagnosis already strongly suspected and with at least some supportive EMG features. In this context, although higher serum NfL levels were highly predictive of a diagnosis of ALS, with positive predictive values 0.92 or above using either cut-off, this appears essentially to confirm clinical judgement. Lower serum NfL values were only weakly predictive of an alternative diagnosis and still compatible with cases of ALS often in the context of relatively slower rates of progression.

Considering the group of patients in whom the diagnosis was uncertain at the initial visit, conclusions are limited by the relatively small number of patients. Nonetheless, our results suggest that very high and very low levels of NfL in this setting might be a useful adjunct to reducing diagnostic delay, with high levels in particular strongly suggesting an active process of neuronal loss.

The setting for our study is not the first point of contact with neurology services for most of the patients. While this would be optimal, for a diagnosis like ALS, with very few cases seen per year by an individual non-specialized neurologist, establishing the value of blood NfL earlier in the diagnostic pathway is currently impractical. Nonetheless, this study indicates the very limited independent role for NfL in making or ruling out a diagnosis of ALS. It may be that NfL may find a more useful role in primary care, where the consistent link to neurological disease activity in the broader sense might usefully triage a range of neurological symptoms by prioritizing those with very high NfL levels. This has been considered analogous to a C-reactive protein test, but for disorders associated with neuronal loss.^21^ Dedicated and very large population-based studies will be needed to test this.

It is also noted that more sensitive means of measuring blood NfL are available, such as single molecule assays.^22^ Although such assays might improve the lower limit of detection, we note that NfL levels in the majority of non-ALS patients in this study were well within the detection range of our platform, and increased assay sensitivity would not be expected to make any difference to the limitations of blood NfL in this setting. Similarly, it is recognized that CSF NfL measurement might offer more sensitivity in relation to CNS pathologies. However, there is a high degree of correlation between CSF and blood levels and,^15,22^ coupled with the significant overlap between more indolent cases of ALS and non-ALS cases seen in this study, this suggests CSF measurement would not alter the diagnostic value in this particular setting.

Our study therefore demonstrates that blood NfL level has a limited role in distinguishing ALS from non-ALS cases independent of clinical judgement, albeit in the specialized clinic setting where EMG and relevant negative neuroimaging will be readily available. A very high NfL level in a case of uncertainty would make ALS more likely and conversely a very low level would make ALS less likely, but some degree of further observation would be needed. Nonetheless, in the former case, it would then perhaps become possible to provide multidisciplinary care at an earlier juncture.

An independently accurate diagnostic biomarker for ALS seems most likely to be related to the TDP-43 proteinopathy that characterizes nearly all cases of ALS.^23^ Meanwhile, the most consistent role of blood NfL currently is in the context of an established diagnosis of ALS, with many studies showing a robust correlation of NfL level with rate of disability progression rate. At a relatively small group level, this already offers the possibility of an earlier marker of therapeutic disease modification.^11^

## Supplementary Material

fcad163_Supplementary_Data

## Data Availability

The data that support the findings of this study are available from the corresponding author upon reasonable request.

## Abbreviations

ALS =: amyotrophic lateral sclerosis
ANOVA =: analysis of variance
CI =: confidence interval
CSF =: cerebrospinal fluid
CV =: coefficient of variation
IQR =: interquartile range
LOD =: limit of detection
LMN =: lower motor neuron
NfL =: neurofilament light chain
NPV =: negative predictive value
PLS =: primary lateral sclerosis
PPV =: positive predictive value
UMN =: upper motor neuron
