# Supplementary material for: Limited value of serum neurofilament light chain in diagnosing amyotrophic lateral sclerosis
Source: Brain Commun. 2023 May 19;5(3):fcad163. doi: 10.1093/braincomms/fcad163 (PMC10244039; doi:10.1093/braincomms/fcad163)
Supplement: fcad163_Supplementary_Data [file fcad163_supplementary_data.docx]

**Supplementary material**

| **Diagnosis** | **N** |
| --- | --- |
| Cramp fasciculation syndrome | 4 |
| Lumbosacral radiculopathy | 3 |
| Hereditary distal motor neuropathy | 1 |
| Kennedy’s disease (SBMA) | 1 |
| Multifocal motor neuropathy | 1 |
| Multiple sclerosis | 1 |
| Cerebral amyloid angiopathy | 1 |
| Functional neurological disease | 1 |
| Parkinson’s disease | 1 |
| Idiopathic sensory axonal neuropathy | 1 |
| Steroid-associated proximal myopathy | 1 |
| Physiological tremor | 1 |
| Late radiation induced brain injury | 1 |
| Pontine stroke | 1 |
| Vocal cord palsy | 1 |
| Hereditary motor neuropathy | 1 |
| Multiple sclerosis | 1 |
| Atypical Guillain-Barre syndrome | 1 |
| Progressive cognitive syndrome | 1 |

**Supplementary Table 1.** List of alternative diagnoses and their frequencies. Below double line are patients in whom it was not possible to determine whether they had ALS or an alternative diagnosis at the first visit but in whom a diagnosis was reached at a subsequent visit.
